# Clusterin accumulates in synapses in Alzheimer’s disease and is increased in apolipoprotein E4 carriers

**DOI:** 10.1093/braincomms/fcz003

**Published:** 2019-06-24

**Authors:** Rosemary J Jackson, Jamie Rose, Jane Tulloch, Chris Henstridge, Colin Smith, Tara L Spires-Jones

**Affiliations:** 1 Centre for Discovery Brain Sciences, UK Dementia Research Institute, University of Edinburgh, Edinburgh, UK; 2 MassGeneral Institute for Neurodegenerative Diseases, Harvard Medical School, Charlestown, MA, USA; 3 Division of Systems Medicine, Neuroscience, Ninewells Hospital & Medical School, University of Dundee, Dundee, UK; 4 Centre for Clinical Brain Sciences, University of Edinburgh, Edinburgh, UK

**Keywords:** Alzheimer, synapse, clusterin, apolipoprotein E, array tomography

## Abstract

One of the major challenges in developing effective therapeutic strategies for Alzheimer’s disease is understanding how genetic risk factors contribute to neurodegeneration. The apolipoprotein epsilon 4 isoform (*APOE4*) and variants in the *Clusterin* (*CLU*) gene (also known as apolipoprotein J) are associated with increased risk of developing Alzheimer’s. Our previous work demonstrated that *APOE4* exacerbates synapse degeneration and synaptic accumulation of toxic oligomeric amyloid beta in human Alzheimer’s and mouse models of disease. Here, we observe clusterin in synapses in human Alzheimer's disease brain. The percentage of synapses containing clusterin is higher in *APOE4* carriers than *APOE3* carriers. Furthermore, we observe oligomeric amyloid beta accumulation within synapses containing clusterin which is also higher in *APOE4* carriers. These data link two genetic risk factors with synapse degeneration in Alzheimer’s and support a potential role for clusterin working with *APOE* in causing synaptic damage.

## Teaser

Jackson and colleagues observe that clusterin accumulates in synapses in human Alzheimer’s disease brain tissue, and that this accumulation is increased in people carrying the Alzheimer’s disease risk gene apolipoprotein E4. These data use high-resolution imaging to link two genetic risk factors with amyloid-mediated synapse degeneration in Alzheimer’s disease.

## Graphical Abstract

**Graphical Abstract:**
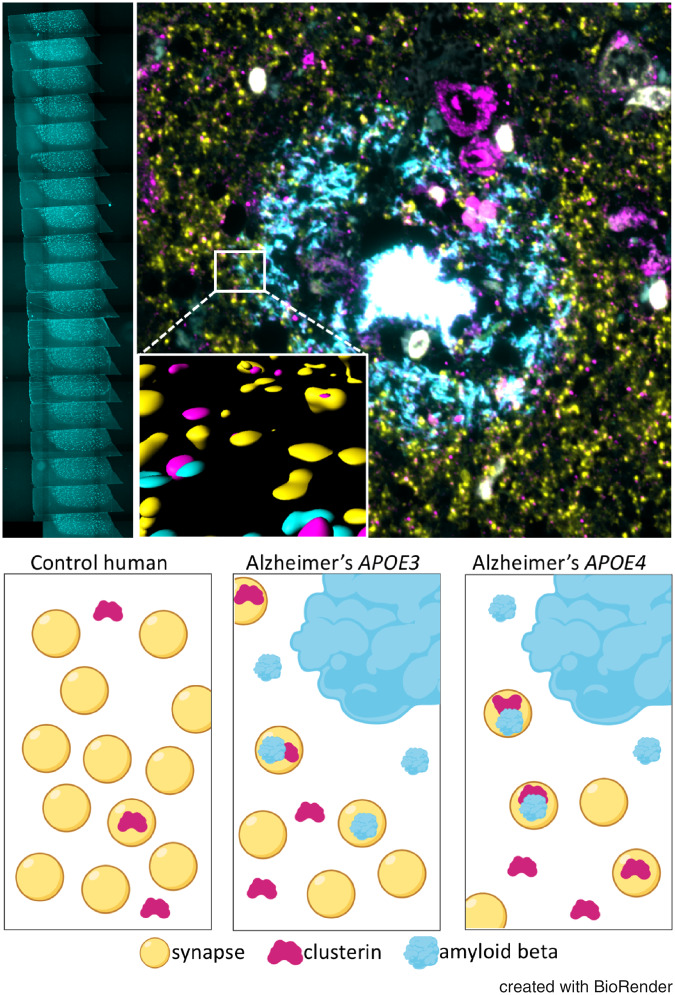

## Introduction

Alzheimer’s disease, a devastating neurodegenerative disease, is characterized neuropathologically by the presence of amyloid beta (Aβ) plaques and tangles made of hyper-phosphorylated and misfolded tau, as well gross neuron and synapse loss in affected areas. Of these hallmarks, it is synapse loss that correlates most strongly with the cognitive decline experienced by people living with Alzheimer's disease (Spires-Jones and Hyman, 2014). Data from animal models of Alzheimer's disease demonstrate that dysfunction of synapses and disrupted synaptic plasticity are key components of neurodegeneration in Alzheimer's disease (Crimins *et al.*, 2013). Investigating the protein changes that underlie the synaptic degeneration caused by Alzheimer's disease is crucial to our understanding of the pathological pathways that are initiated by this disease.

Although most cases of Alzheimer's disease are not directly heritable, genetic risk factors have been identified, the strongest of which is the epsilon 4 isoform of apolipoprotein E (*APOE4*). The *APOE4* allele has been shown to increase the risk of Alzheimer's disease in a dose-dependent manner when compared with the more common *APOE3* allele whereas the rarer *APOE2* allele is protective (Corder *et al.*, 1993; Corder and Roses, 1996). The possession of two copies of *APOE4* has been shown to not only increase the chance of getting Alzheimer's disease by 12-fold that of a person with two copies of *APOE3*, but also lower the average age of clinical onset to 68 years of age down from 84 (Corder *et al.*, 1993). *APOE4* has also been shown to affect the speed of cognitive decline in both Alzheimer's disease individuals as well as having an effect on the cognitive function of non-demented older adults (Schiepers *et al.*, 2012; Lim *et al.*, 2015).

Our previous work has shown that in both a mouse model of Alzheimer's disease and human post-mortem tissue from Alzheimer's disease individuals synapse density is decreased inside the Aβ plaque core and the Aβ oligomer containing halo. This loss of synapse density returns to near control levels when greater than 35 μm from the plaque halo (Koffie *et al.*, 2009, 2012). Furthermore, we saw a significantly lower density of synapses near plaques in *APOE4* carriers compared with *APOE3* carriers and the density of synapses far from plaques was also significantly lower (Koffie *et al.*, 2012). We have recently completed a systematic literature search and comprehensive review of the published papers relating ApoE to neurodegeneration, inflammation and/or the spread of pathological proteins through the brain (Tzioras *et al.*, 2019). The review of the 108 papers that matched our inclusion criteria shows strong evidence in the field implicating *APOE4* in synapse degeneration, neurodegeneration and inflammation and highlighted the need for a better understanding of the mechanisms through which *APOE* affects synapse degeneration. One potential mechanism leading from *APOE4* to synapse loss is via interacting with other genetic risk factors to influence accumulation of toxic oligomers of Aβ in synapses.

Genome wide association studies over the past decade uncovered additional risk factors to *APOE4* including *TREM2*, *PICALM* and *CLU* (Harold *et al.*, 2009; Guerreiro *et al.*, 2013). Clusterin, also called apolipoprotein J, is the second most abundant apolipoprotein in the human brain and has crucial roles in trafficking and clearance of Aβ similar to the role played by ApoE (Nuutinen *et al.*, 2009). Indeed, there are many parallels between clusterin and ApoE that make an interaction between them of interest, not least of which is that both have been shown to act as a modulator of multiple pathways of interest during diseases and aging including; glucose metabolism, transport of cholesterol, amyloid beta trafficking and regulation of the immune system (Hakkoum *et al.*, 2008; Park *et al.*, 2014; Foster *et al.*, 2019).

In this study, we tested the hypothesis that clusterin and ApoE4 work together to cause synapse degeneration in Alzheimer's disease by increasing accumulation of oligomeric amyloid beta at synapses. To achieve this, we isolated synaptoneurosomes from post-mortem tissue of Alzheimer's disease and non-demented control individuals of known *APOE* genotypes which were then probed for clusterin levels. We also used the high-resolution imaging technique array tomography to further assess the impact of ApoE4 and Alzheimer's disease on the synaptic localization of Aβ and clusterin and the related impact on the loss of synaptic density in Alzheimer’s disease. We observe accumulation of clusterin in synapses which is higher in *APOE4* carriers than *APOE3* carriers.

## Materials and methods

All materials were purchased from Sigma-Aldrich unless otherwise stated.

### Human subjects

Tissue from superior temporal cortex, Brodmann Area 41/42 of human subjects with Alzheimer’s disease or no neurological phenotype was used for this study. Table 1 shows characteristics of the human subjects.

**Table 1:** Human subject characteristics

Case ID	Diagnosis	APOE genotype	age (range)	sex (m,f)	Braak stage	PMI (hours ± stdev)
**cases used for western blot**						
20122	non-demented control	3/3	59	M	0	74
14395	non-demented control	3/3	74	F	0	41
19686	non-demented control	3/3	77	F	I	75
22612	non-demented control	3/3	61	M	0	70
001.26475	non-demented control	3/3	78	M	I	39
24340	non-demented control	3/3	53	M	0	53
001.28406	non-demented control	3/3	79	M	II	72
001.28793	non-demented control	3/3	79	F	II	72
001.28402	non-demented control	3/3	79	M	I	49
**GROUP**	**n=9**	** **	**59 (53-79)**	**6,3**	**I**	**74 ± 15**

15221	non-demented control	4/3	53	M	0	114
15809	non-demented control	4/3	58	M	0	90
20593	non-demented control	4/3	60	M	0	52
16425	non-demented control	4/3	61	M	0	99
001.2555	non-demented control	4/3	74	M	0	66
22629	non-demented control	4/3	59	F	0	53
**GROUP**	**n=6**	** **	**61 (53-74)**	**5,1**	**0**	**79 ± 26**

19994	Alzheimer’s disease	3/3	87	F	VI	89
15258	Alzheimer’s disease	3/3	65	M	VI	80
001.19595	Alzheimer’s disease	3/3	87	M	VI	58
001.28410	Alzheimer’s disease	3/3	62	F	VI	109
22223	Alzheimer’s disease	3/3	87	F	IV	83
24527	Alzheimer’s disease	3/3	81	M	V	74
001.28771	Alzheimer’s disease	3/3	85	M	VI	91
001.28785	Alzheimer’s disease	3/3	78	F	No stageing	76
**GROUP**	**n=8**		**79 (62-87)**	**4,4**	**VI**	**83 ± 15**

10591	Alzheimer’s disease	4/3	86	M	VI	76
15810	Alzheimer’s disease	4/3	73	F	VI	96
001.15811	Alzheimer’s disease	4/3	81	F	VI	41
15259	Alzheimer’s disease	4/3	87	F	VI	28
23394	Alzheimer’s disease	4/3	88	F	V	59
001.24322	Alzheimer’s disease	3/4	80	M	VI	101
24526	Alzheimer’s disease	3/4	79	M	VI	65
19690	Alzheimer’s disease	3/4	57	M	VI	58
001.25739	Alzheimer’s disease	3/4	85	F	VI	45
001.26718	Alzheimer’s disease	3/4	78	M	VI	74
001.26732	Alzheimer’s disease	3/4	76	M	VI	66
**GROUP**	**n=11**	** **	**79 (57-88)**	**6,5**	**VI**	**64 ± 22**

**cases used for array tomography**						

001.28406	non-demented control	3/3	79	M	II	72
001.26495	non-demented control	3/3	78	M	I	39
001.28793	non-demented control	3/3	79	F	II	72
19686	non-demented control	3/3	77	F	I	75
001.28797	non-demented control	3/3	79	M	0	57
**GROUP**	**n=5**	** **	**78 (77-79)**	**3,2**	**I**	**63 ± 15**

1424	Alzheimer’s disease	3/3	89	M	VI	25
001.28410	Alzheimer’s disease	3/3	62	F	VI	109
001.28771	Alzheimer’s disease	3/3	85	M	VI	91
24527	Alzheimer’s disease	3/3	81	M	V	74
**GROUP**	**n=4**		**79 (62-89)**	**3,1**	**VI**	**74 ± 36**

001.25739	Alzheimer’s disease	4/3	85	F	VI	45
001.26718	Alzheimer’s disease	4/3	78	M	VI	74
24526	Alzheimer’s disease	4/3	79	M	VI	65
19690	Alzheimer’s disease	4/3	57	M	VI	58
001.26732	Alzheimer’s disease	4/3	76	M	VI	66
001.29521	Alzheimer’s disease	4/3	95	M	VI	96
001.29135	Alzheimer’s disease	4/3	90	M	VI	73
**GROUP**	**n=7**	** **	**80 (57-95)**	**6,1**	**VI**	**68 ± 16**

Use of human tissue for post-mortem studies was reviewed and approved by the Edinburgh Brain Bank ethics committee and the medical research ethics committee (the Academic and Clinical Central Office for Research and Development, a joint office of the University of Edinburgh and NHS Lothian, approval number 15-HV-016). The Edinburgh Brain Bank is a Medical Research Council funded facility with research ethics committee (REC) approval (11/ES/0022).

### *APOE* genotyping

DNA was extracted from ∼25 mg of cerebellum tissue sampled from each case using the QIAamp DNA mini kit (Qiagen, Hilden, Germany) according to manufacturer’s instructions. Polymerase chain reaction (PCR) was performed on the extracted DNA. PCR using Master mix (Promega, Madison, WI) and contained 1 μM of primer and 10% DMSO. The forward primer was 5ʹ-taagcttggcacggctgtccaagg-3ʹ and the reverse primer 5ʹ-acagaattcgccccggcctggtacactgcc-3ʹ. Pure *APOE* ε2, *APOE* ε3 and *APOE* ε4 DNA were amplified by PCR to use as positive controls. The reactions were then heated to 94°C for 10 min before being cycled 32 times in a thermal cycler (Thermo Fisher Scientific, Waltham, MA). The cycle consisted of 30 s of denaturing at 94°C, 30 s of annealing at 56°C and then 1 min of elongation at 72°C.

The product from PCR was then digested with the restriction enzyme HhaI (New England Biolabs, Ipswich, MA) overnight at 37°C. Digested DNA was then loaded onto Novex TBE 20% pre-cast gels with 15 wells (Thermo Fisher Scientific, Waltham, MA) and separated by size using electrophoresis for 2 h at 200 V. Gels were then removed from the cassette incubated with SYBR safe DNA Gel Stain (Thermo Fisher Scientific, Waltham, MA) and visualized using UV light on a gene genius bio imaging system (Syngene, Cambridge, UK). The banding pattern indicated *APOE* genotype.

### Synaptoneurosome isolations and western blotting

Synaptoneurosomes and crude homogenate preparations were biochemically isolated according to Tai *et al.* (2012). Approximately 200 mg of fresh-frozen brain tissue from human Alzheimer's disease and control temporal cortex was homogenized in a glass homogenizer with 1 ml ice-cold buffer A (5 mmol/L KCl, 1 mmol/L MgCl_2_, 25 mmol/L HEPES pH 7.5, 120 mmol/L NaCl and 2 mmol/L CaCl_2_), supplemented with 2 mmol/L DTT, protease inhibitors (cOmplete mini, Roche, Basel, Switzerland), and phosphatase inhibitors (Millipore, Billerica, MA). Homogenates were filtered through two layers of 80-μm nylon filters (Millipore, Billerica, MA). A solution of 200 μl of this filtered homogenate was mixed with 200 μl water and 70 μl 10% sodium dodecyl sulphate (SDS) and boiled to prepare the crude homogenate fraction.

To prepare synaptoneurosomes, the remainder of the filtered homogenate was passed through a 5-μm membrane filter (Durapore, Millipore, Billerica, MA) before being centrifuged at 1000 G for 10 min. The supernatant was removed, and the pellet resuspended in 200 μl buffer A and centrifuged again at 1000 G for 5 min. The supernatant was discarded and the synaptoneurosome pellets were snap frozen on dry ice and then transferred to −80°C.

Synaptoneurosome pellets were resuspended in 400 μl of Buffer B (50 mmol/L Tris [pH 7.5], 1.5% SDS and 2 mmol/L DTT) and boiled for 5 min. 10% SDS was added to the supernatant fraction to bring it up to 1.5% SDS and this was also boiled for 5 min to prepare for western blotting. Protein concentration in each sample was estimated by bicinchoninic acid (BCA) assay.

Synaptoneurosomes or crude homogenate preparations were loaded onto 15 well NuPAGE 4–12% Bis-Tris precast polyacrylamide gels (Invitrogen, Paisley, UK) along with molecular weight marker (Li-Cor, Cambridge, UK). Proteins were transferred to nitrocellulose membranes (Bio-Rad, Hemel Hempstead, UK), which were probed with the following primary antibodies: β-actin (ab8226, Abcam, 1:2000), Synaptophysin (ab8049, Abcam, 1:10 000), GAPDH (ab8245, Abcam, 1:2000), Histone H3 (1:1000, ab1791, Abcam), PSD95 (1:1000, D27E11, Cell Signaling Technology) and clusterin (1:500, sc-8354, Santa Cruz Biotechnology). After rinsing off unbound primary antibodies, membranes were incubated with 680 and 800 IR dye labelled secondary antibodies (1:50 000, LI-COR Biosciences) and visualized on an odyssey infra-red system (LI-COR Biosciences).

### Array tomography

Tissue from the BA41/42 area of the cortex was embedded as described in Kay *et al.* (2013). In brief, post-mortem samples were collected at autopsy, cut into small cortical blocks and fixed in 4% paraformaldehyde for 2–3 h. Samples were then dehydrated in ascending concentrations of ethanols and incubated in LR white resin overnight. Cortical blocks were then baked in LR white resin which was polymerized at 56°C for 24 h. Tissue blocks were sectioned into ribbons of 70 nm serial sections which were collected on gelatin-coated coverslips. The ribbons were stained with primary and secondary antibodies and the same region of interest imaged in each section of the ribbon. Antibodies from the first day of staining were eluted off of the ribbon in stripping buffer (0.2 m NaOH, 0.02% SDS in dH_2_O) then ribbons were re-probed with a second set of antibodies and images were taken in the same regions of interest as day 1. The primary antibodies on day 1 was 1C22 [1:50, kind gift of Dominic Walsh (Yang *et al.*, 2015)]. Primary antibodies used on day 2 were mouse anti-synaptophysin (1:50, ab8049, Abcam), and goat anti-clusterin (1:50, sc-6420, Santa Cruz Biotechnologies). The Alexa Fluor conjugated secondary antibody used on day 1 was donkey anti-mouse 594 (Invitrogen). Secondary antibodies used on day 2 were donkey anti-mouse 594 (A31571) and donkey anti-goat 594 (A11058, Invitrogen). All secondary antibodies were used at a 1:50 dilution and DAPI was used on both days to label nuclei. Images were acquired with a Zeiss Axio Imager Z2 upright microscope equipped with a CoolSnap digital camera using a 63× oil objective. Images were acquired from the same location on each serial section of the ribbon. Image stacks were aligned and regions of interest in the neuropil selected in image J using custom macros and the multistack reg plugin (Thevenaz *et al.*, 1998; Micheva *et al.*, 2010). Custom MATLAB scripts were used to detect synaptic puncta and determine whether clusterin and/or Aβ staining was present in each synapse.

### Statistical analysis

The experimental unit for all experiments was a mean or median for each subject. The experimenter was blind to condition during image analysis. Numbers of subjects in each group can be found in Table 1. Statistics were calculated using graph pad prism (version 7.0c). Normality was tested with the Shapiro–Wilk normality test. For parametric data (western blots and synapse density) one or two-way ANOVA was used to compare groups followed by Tukey’s *post hoc* tests between individual pairs of groups. Co-localization data of the percentage of synapses containing Aβ, clusterin or both were not normally distributed thus were analysed with Kruskal–Wallis tests followed by *post hoc* Mann–Whitney *U* test between pertinent groups.

### Data availability

Data from Western blot experiments for each case can be found in Supplementary Table 1, and data from array tomography experiments can be found in Supplementary Table 2. Raw images are available from the corresponding author upon request. Custom image analysis macros for array tomography can be downloaded from https://doi.org/10.7488/ds/2268

## Results

### Clusterin is increased in the Alzheimer's disease *APOE4* synapse but not in crude homogenate

**Figure 1:**
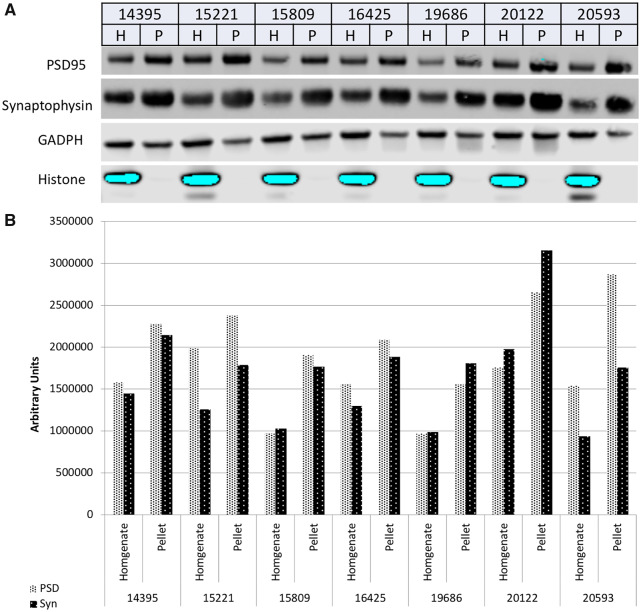
**Preparation of synaptoneurosomes. Crude homogenates (H) and synaptoneurosomes pellets (P) were isolated from temporal cortex of Alzheimer's disease and control subjects.** Representative western blots (**A**) and quantification of synaptic proteins (**B**) is shown for seven cases (indicated by their BBN ID numbers 14395, 15221, 15809, 16425, 19686, 20122 and 20593). Enrichment of synaptic proteins (synaptophysin and PSD95) and exclusion of nuclear histone protein in the synaptoneurosome pellets compared with the homogenates was confirmed for each case included in the study. GAPDH is used to ensure equal protein loading.

To investigate the effects of ApoE4 on the amount of clusterin in the synapse, synaptoneurosomes were prepared using a previously described method (Tai *et al.*, 2012). The synaptically enriched fraction was probed for synaptophysin (for pre-synapses) and PSD95 (for post-synapses) to ensure enrichment of synaptic elements as well as histone H3 to ensure exclusion of a nuclear marker (Fig. 1). Samples which contained histone or did not show enrichment of the synapse were either remade or discarded from the study.

To determine the quality of protein in our human brain samples, we assessed protein degradation using the ‘HUSPIR’ ratio or (Bayés *et al.*, 2014). To find this value, synaptoneurosmes were run on western blot and probed with an antibody against NMDAR2B antibody. This recognizes two to three bands, the full length protein at 170 kDa and degradation products at 150 kDa and below. This degradation product is only found post-mortem and not in autopsy tissue thus comparing these two bands is a good indication of post-mortem protein integrity. Samples with a degradation ratio of less than 1 were excluded from the study (data not shown).

**Figure 2:**
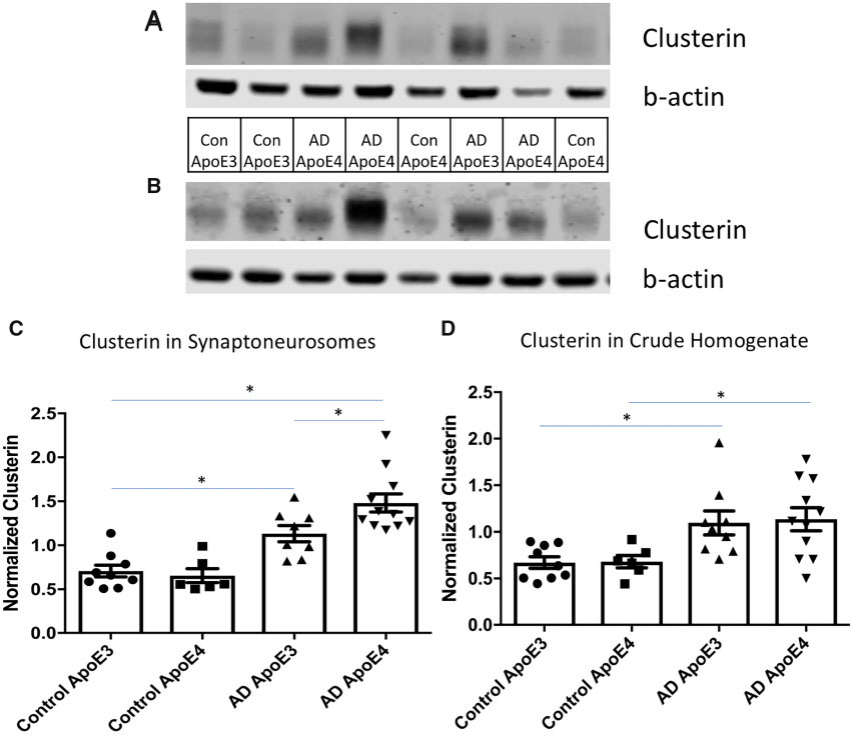
**Clusterin is increased in the synaptic compartment in Alzheimer's disease with highest levels in *APOE4* carriers.** Western blot (**A**, **B**) analysis shows an increased level of clusterin when comparing Control with Alzheimer's disease in both Crude Homogeante (**A**, **C**) and in Synaptically enriched preps (**B**, **D**). In synapses, there is a further increase in the Alzheimer's disease *APOE4* cases compared with Alzheimer's disease *APOE3* cases (*Tukey’s *post hoc* tests, *P* < 0.05).

Western blot analysis of homogenate of temporal cortex from post-mortem brain (Fig. 2A, un-cropped western blots are shown in Supplementary Figs 1 and 2) shows an increase in the amount of clusterin in Alzheimer's disease compared with control, with no effect of *APOE* genotype on this increase (two-way ANOVA effect of disease *F* (1, 30) = 16.96, *P* = 0.0003, effect of *APOE* genotype *F* (1, 30) < 0.0001, *P* = 0.998; Tukey’s *post hoc* between control *APOE3* and Alzheimer's disease *APOE3 P* = 0.0241, Tukey’s *post hoc* between Control *APOE4* and Alzheimer's disease *APOE4 P* = 0.0421, Fig. 2C). This is consistent with previous studies, although a recent study indicated that clusterin is increased in Alzheimer's disease due in part to its interaction with Aβ plaques (Miners *et al.*, 2017). As Aβ plaques are more prevalent in individuals with an *APOE4* genotype one might expect a slight increase in the amount of clusterin in Alzheimer's disease *APOE4* cases compared with Alzheimer's disease *APOE3* cases (Rebeck *et al.*, 1993). However, this is not seen in our cases, potentially because any effect of *APOE4* on clusterin is diluted by the rest of the cellular milieu. In contrast, western blot analysis of synaptoneurosomes shows an increase in clusterin in Alzheimer's disease cases (Fig. 2B, two-way ANOVA effect of disease *F* (1, 30) = 44.24, *P* < 0.0001, effect of *APOE* genotype *F* (1, 30) = 2.48, *P* = 0.126, interaction *F* (1, 30) = 4.551, *P* = 0.0412, Tukey’s *post hoc* between control *APOE3* versus Alzheimer's disease *APOE3 P* = 0.0137 and Tukey’s *post hoc* between control *APOE4* versus Alzheimer's disease *APOE4 P* < 0.0001) and a further increase in the Alzheimer's disease *APOE4* cases compared with Alzheimer's disease *APOE3* (Tukey’s *post hoc P* = 0.0413, un-cropped blots are shown in Supplementary Fig. 2). This indicates that an *APOE4* genotype increases the amount of clusterin at remaining synapses in Alzheimer's disease but not overall in the temporal cortex. Further analysis shows that within the Alzheimer's disease *APOE4* cases, more than in any other group, the amount of clusterin found at the synapse is higher than the amount found in the crude homogenate from the same case, indicating that clusterin is increased specifically in the synapse in these cases (Supplementary Fig. 3). Analysis of western blot data for each case can be found in Supplementary Table 1.

### *APOE4* is associated with exacerbated synaptic loss and increased synaptic Aβ co-localization in Alzheimer’s disease

**Figure 3:**
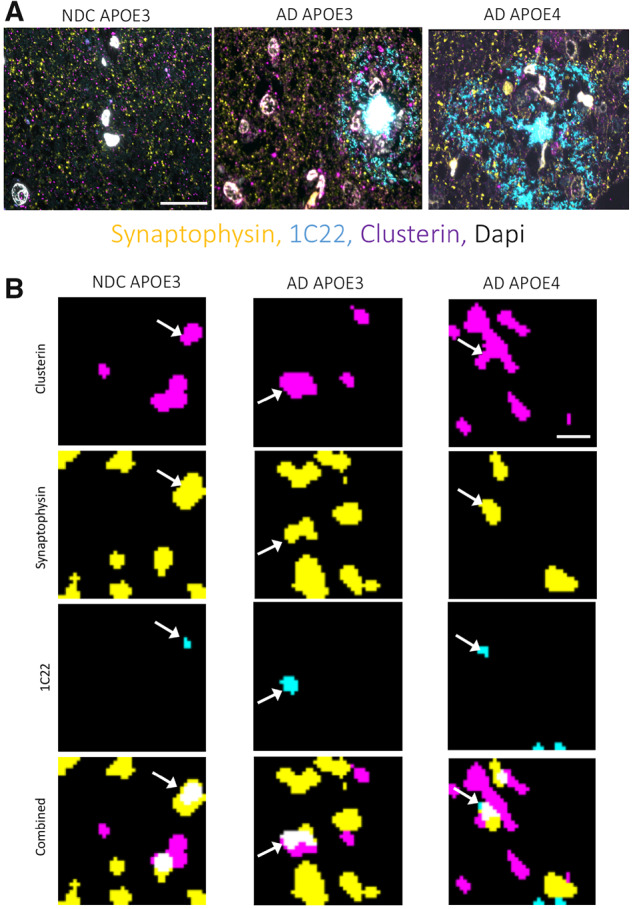
**Representative images of array tomography staining.** Array tomography ribbons from non-demented controls (NDC), Alzheimer's disease *APOE3* and Alzheimer's disease *APOE4* individuals were stained for pre-synapses (synaptophysin, yellow), oAβ (1C22, cyan) and clusterin (magenta). Images shown in (**A**) are maximum intensity projections of four serial sections (aligned raw images). Images shown in (**B**) are maximum intensity projections of two serial sections from analysed image stacks that have been thresholded and single section noise removed in MATLAB. Each channel is shown separately with the merge in the bottom image. Arrows indicate synapses containing both clusterin and oAβ staining. Scale bars represent 15 μm (**A**) and 1 μm (**B**).

**Figure 4:**
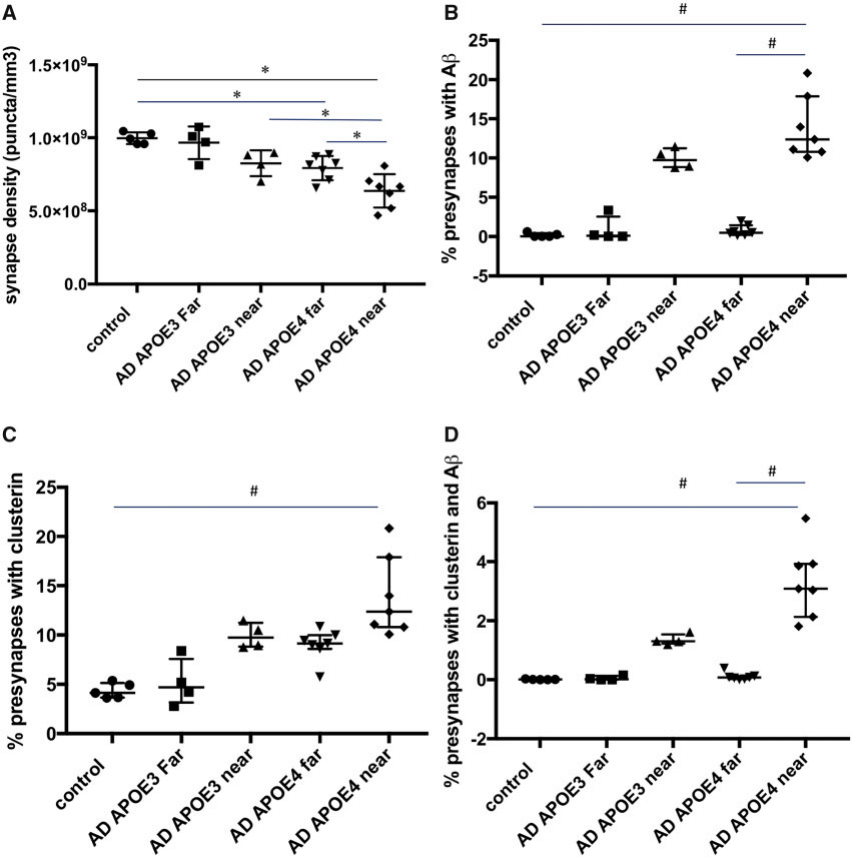
**Analysis of synaptic punctate by array tomography.** (**A**)There is a significant decrease in the pre-synaptic density in Alzheimer’s cases near plaques (<10 μm) compared with far from plaques (>45 μm) which is exacerbated by *APOE4* genotype (one-way ANOVA *F* [4, 22] = 14.2, *P* < 0.0001). (**B**) There is also a significant increase in the percent of Aβ (1C22) positive synapses near plaques compared with far from plaques that is most pronounced in E4 cases (Kruskal–Wallis test, *P* = 0.0003). (**C**) Clusterin in pre-synaptic terminals is increased in Alzheimer's disease *APOE4* carriers (Kruskal–Wallis, *P* = 0.0003). (**D**) Similarly, the percentage of synapses containing both clusterin and Aβ is highest in Alzheimer's disease *APOE4* carriers near plaques carriers (Kruskal–Wallis, *P* = 0.0002). (*P < 0.05, **P < 0.01, ****P < 0.0001 Tukey’s *post hoc* test. ^#^*P* < 0.05, ^##^*P* < 0.01, ^###^*P* < 0.001 *post hoc* Mann–Whitney *U*.) Each symbol represents the mean for a single case in (**A**) with error bars representing standard deviation. Each symbol in (**B–F**) represents the median for a single case with the error bars showing inter-quartile ranges.

While western blots allow measurements of protein abundance in synaptoneurosomes, they do not provide detailed information about synapse loss, specific changes in synapses near plaques or the co-localization of multiple proteins within synapses. Thus to test the hypothesis that *APOE* genotype affects the synaptic accumulation of clusterin and Aβ, we used the high-resolution array tomography technique (Micheva *et al.*, 2010), which we have optimized for use in human post-mortem tissue (Koffie *et al.*, 2012; Kay *et al.*, 2013). Using this technique, we can observe the co-localization of clusterin and Aβ within individual pre-synaptic terminals as well as examining synapse density (Fig. 3). Due to limited sample availability of tissue prepared at autopsy for this specialized technique, we did not have enough subjects for a control *APOE4* group. In a previous study using array tomography, we observed that *APOE4* exacerbates synapse loss near plaques and is associated with increases in the percentage of synapses containing Aβ (Koffie *et al.*, 2012). Here, we replicate these findings in a different set of subjects (Fig. 4A and B), and further examine clusterin and Aβ in synapses. Regions of interest in the neuropil were selected from image stacks and divided into two groups, those which were near plaques (<10 μm from plaque edge) and those far from plaques (>45 μm from plaque edge). Synaptophysin was used as a marker of pre-synaptic terminals. Consistent with our previous study (Koffie *et al*., 2012), we observe a loss of synapses in Alzheimer's disease compared with control brain, the loss being exacerbated near plaques in *APOE4* cases (one-way ANOVA, *F*(4.22) = 14.2, *P* < 0.0001, *post hoc* Tukey’s tests Alzheimer's disease *APOE4* near versus far from plaques *P* = 0.032, control versus Alzheimer's disease *APOE4* near *P* < 0.0001). Further, there is a significant drop in pre-synaptic density near plaques when compared with far from plaques in Alzheimer's disease cases (two-way ANOVA effect of plaque distance, *F* (1, 18) = 11.21, *P* = 0.0036) and a significant effect of *APOE* genotype on synapse density (two-way ANOVA effect of *APOE* genotype, *F* (1, 18) = 16.67, *P* = 0.0007, Fig. 4A). Also consistent with previous data was the finding that synapses near plaques were significantly more likely to co-localize with Aβ (Fig. 4B, Kruskal–Wallis test χ^2^(4) = 21.18, *P* = 0.0003, *post hoc* Mann–Whitney *U* between Alzheimer's disease *APOE3* near and *APOE3* far *P* = 0.0286 and between Alzheimer's disease *APOE4* near and *APOE4* far *P* = 0.0006) and that this increase was greater in *APOE4* Alzheimer's disease cases (*post hoc* Mann–Whitney *U* between Alzheimer's disease *APOE3* near versus Alzheimer's disease *APOE4* near *P* = 0.0061). Analysis of array tomography data for each case can be found in Supplementary Table 2.

### *APOE4* is associated with an increased synaptic co-localization of clusterin and increased synaptic co-localization of clusterin and oligomeric Aβ

Array tomography was used to investigate whether the increase in clusterin seen in the synaptoneurosome using proteomics and western blot was associated with accumulation of clusterin and Aβ within the same synapses.

When the percentage of synapses co-localizing with clusterin was examined, we observed an increase in Alzheimer’s disease cases compared with controls with the largest effect near plaques in *APOE4* carriers (Fig. 4C, Kruskal–Wallis test χ^2^(4) = 21.16, *P* = 0.0003; *post hoc* Mann–Whitney *U* between Alzheimer's disease *APOE3* near and *APOE3* far *P* = 0.0286, between Alzheimer's disease *APOE4* near and *APOE4* far *P* = 0.0023, between Alzheimer's disease *APOE3* far and Alzheimer's disease *APOE4* far *P* = 0.012, and between control and *APOE4* far *P* = 0.0025).

One of the benefits of array tomography is the ability to strip antibodies and re-probe the same tissue ribbon with different antibodies thus allowing the analysis of the co-localization of several protein markers to the same synapse. We therefore looked at the co-localization of clusterin and Aβ together at the synapse to see if Alzheimer's disease *APOE4* cases were more likely to have synapses positive for both markers. There is an increase in the percentage of synaptic puncta co-localizing with both Aβ and clusterin in Alzheimer's disease *APOE4* cases near plaques (Fig. 4D, Kruskal–Wallis test χ^2^(4) = 21.61, *P* = 0.0002, *post hoc* Mann–Whitney *U* between Alzheimer's disease *APOE3* near and *APOE3* far *P* = 0.0286, between Alzheimer's disease *APOE4* near and *APOE4* far *P* = 0.0006 and between Alzheimer's disease *APOE3* near and Alzheimer's disease *APOE4* near *P* = 0.0061).

## Discussion

The effects of ApoE in Alzheimer's disease have long been studied but by comparison the study of the related apolipoprotein clusterin has been limited despite it too being a genetic risk factor for the disease. Here, we show by multiple systems in post-mortem tissue that an *APOE4* genotype affects the concentration of clusterin specifically in the synapse and but not in the brain homogenate overall. Moreover, this increase in clusterin is found to coincide with an increase in the amount of Aβ in the same synapses.

As the second most abundantly expressed apolipoprotein in the brain, clusterin performs many of the same roles as ApoE including the transport of cholesterol and other necessary lipids to the neuron (Dong *et al.*, 2017), thus it is possible that changes in clusterin and ApoE may be related. An *APOE4* genotype has been shown to have an effect on the amount of ApoE in the brain due in part to the increased speed with which ApoE4 is degraded (Riddell *et al.*, 2008). When *Apoe* is knocked out of a mouse line there is an increase in the amount of clusterin mRNA indicating that clusterin can compensate for the loss of ApoE (Stone *et al.*, 1998). Further highlighting a possible compensatory mechanism between ApoE and clusterin is the study by DeMattos *et al.* (2004) which showed only slight changes in plaque load and deposition in PDAPP mice when either *Clusterin* or *Apoe* was knockout but a much greater effect in the mice where both were knocked out.

The increase in clusterin at the synapse which we observed could be due to a number of factors including neuronal stress. It is known that stress, and specifically Aβ induced stress, results in an increase in intracellular clusterin and a decrease in extracellular clusterin (Killick *et al.*, 2014). A stress response could help explain the increase in the amount of clusterin in Alzheimer’s disease. Adding to this hypothesis is a study showing that clusterin is up-regulated in the cerebrospinal fluid (CSF) of Alzheimer's disease individuals and that higher CSF clusterin was associated with an increase in entorhinal cortex atrophy (Desikan *et al.*, 2014). Although this increase in CSF could be a response to neuron degradation rather than a causative factor it does lead to the question of whether clusterin is involved in Aβ-mediated synapse loss. A question raised by our data is whether an increase in clusterin has a protective or a detrimental effect on the synapse. Clusterin can bind Aβ, prevent fibrillization of Aβ, and prevent the loss of long-term potentiation and memory when injected into a rat, which points towards a protective role for clusterin in Alzheimer's disease (Cascella *et al.*, 2013). On the other hand, knockout of clusterin in rat primary neurons prevented Aβ-induced neuronal death which indicates the detrimental effects of Aβ may be at least in part due to clusterin (Killick *et al.*, 2014). However, it is worth noting the limitations of these cell death data since supraphsyiological levels of Aβ are required to induce cell death *in vitro*. Furthermore *in vivo*, pathological tau, not Aβ, is thought to drive cell death. *In vitro* studies have shown that incubation of Aβ fibrils and Aβ oligomers with clusterin causes decreased uptake of both fibrils and oligomers by microglia and just oligomers by astrocytes (Mulder *et al.*, 2014). This could lead to the increased deposition of Aβ as described by DeMattos *et al.*, but could also increase the amount of Aβ bound to apolipoproteins which could then target that Aβ to the synapse causing an increase in synapse degeneration.

Clusterin is known to bind Aβ in the extracellular space and prevent fibrillization (Cascella *et al.*, 2013). This could indicate that the increase in Aβ and clusterin that we observe at the synapse is due to an internalization of clusterin bound to Aβ. However, other studies have shown that intracellular clusterin is increased following Aβ application indicating that up-regulation of intracellular clusterin is a response to Aβ accumulation in the synapse (Killick *et al.*, 2014). Of course, it is possible that both occur in the cell and that cellular stress caused by Aβ at the synapse causes internalization of clusterin bound to Aβ causing a positive feedback loop. Recent work in APP/PS1 mice crossed with clusterin knockout mice demonstrates that the loss of clusterin shifts amyloid deposition to cerebrovasculature due to a shift in clearance to perivascular drainage pathways (Wojtas *et al.*, 2017). It is possible that our observed increased localization of clusterin in synapses in Alzheimer’s disease may have implications beyond local synaptic protein changes. Shifting subcellular clusterin localization could potentially impact on the wider neuro-glial vascular unit, but this requires further investigation.

It is possible that, similar to ApoE, clusterin has some effects by acting via Aβ but other effects via downstream interactor molecules such as TREM2. These downstream effects may influence the cellular milieu of the Alzheimer's disease brain and be of greater importance to the overall neurodegeneration than the effects on Aβ alone. It is entirely likely that clusterin has multiple roles in the brain and that these jobs depend in part upon subcellular localization of the protein. To fully understand the implications of this study, further investigation of the role of this important risk factor on the synaptic changes associated with Aβ and Alzheimer's disease is required. The data presented here from human post-mortem brain tissue highlight the importance of studying synaptic effects of clusterin and its interactions with pathological proteins in Alzheimer’s disease.

## Supplementary Material

fcz003_Supplementary_Data

## Abbreviations

AD: Alzheimer’s Disease
Aβ: amyloid beta
*APOE* for gene, ApoE for protein: Apolipoprotein E
